# Osteoporosis Risk in Hemodialysis Patients: The Roles of Gender, Comorbidities, Biochemical Parameters, Health and Diet Literacy

**DOI:** 10.3390/nu14235122

**Published:** 2022-12-02

**Authors:** Lan T. H. Le, Loan T. Dang, Tsae-Jyy Wang, Tuyen G. Do, Dung H. Nguyen, Trung A. Hoang, Minh D. Pham, Binh N. Do, Hoang C. Nguyen, Tu T. Tran, Linh V. Pham, Lien T. H. Nguyen, Hoi T. Nguyen, Nga T. Trieu, Thinh V. Do, Manh V. Trinh, Tung H. Ha, Dung T. Phan, Shwu-Huey Yang, Ngoc N. M. Le, Kien T. Nguyen, Tuyen Van Duong

**Affiliations:** 1 Training and Direction of Healthcare Activity Center, Thai Nguyen National Hospital, Thai Nguyen City 241-24, Vietnam; 2 Biochemistry Department, Thai Nguyen National Hospital, Thai Nguyen City 241-24, Vietnam; 3 Director Office, Thai Nguyen National Hospital, Thai Nguyen City 241-24, Vietnam; 4 School of Nursing, National Taipei University of Nursing and Health Sciences, Taipei 112-19, Taiwan; 5 Faculty of Nursing and Midwifery, Hanoi Medical University, Hanoi 115-20, Vietnam; 6 Hemodialysis Department, Nephro-Urology-Dialysis Center, Bach Mai Hospital, Hanoi 115-19, Vietnam; 7 Department of Nutrition, Military Hospital 103, Hanoi 121-08, Vietnam; 8 Department of Nutrition, Vietnam Military Medical University, Hanoi 121-08, Vietnam; 9 Department of Military Science, Vietnam Military Medical University, Hanoi 121-08, Vietnam; 10 Department of Infectious Diseases, Vietnam Military Medical University, Hanoi 121-08, Vietnam; 11 President Office, Thai Nguyen University of Medicine and Pharmacy, Thai Nguyen City 241-17, Vietnam; 12 International Ph.D. Program in Medicine, College of Medicine, Taipei Medical University, Taipei 110-31, Taiwan; 13 Department of Internal Medicine, Thai Nguyen University of Medicine and Pharmacy, Thai Nguyen 241-17, Vietnam; 14 Department of Pulmonary & Cardiovascular Diseases, Hai Phong University of Medicine and Pharmacy Hospital, Hai Phong 042-12, Vietnam; 15 President Office, Hai Phong University of Medicine and Pharmacy, Hai Phong 042-12, Vietnam; 16 Director Office, Hai Phong International Hospital, Hai Phong 047-08, Vietnam; 17 Hemodialysis Division, Hai Phong International Hospital, Hai Phong 047-08, Vietnam; 18 Director Office, Bai Chay Hospital, Ha Long 011-21, Vietnam; 19 Director Office, Quang Ninh General Hospital, Ha Long 011-08, Vietnam; 20 Director Office, General Hospital of Agricultural, Hanoi 125-16, Vietnam; 21 Faculty of Nursing, Hanoi University of Business and Technology, Hanoi 116-22, Vietnam; 22 Nursing Office, Thien An Obstetrics and Gynecology Hospital, Hanoi 112-06, Vietnam; 23 School of Nutrition and Health Sciences, Taipei Medical University, Taipei 110-31, Taiwan; 24 Nutrition Research Center, Taipei Medical University Hospital, Taipei 110–31, Taiwan; 25 Research Center of Geriatric Nutrition, Taipei Medical University, Taipei 110–31, Taiwan; 26 College of Medicine, Hanoi Medical University, Hanoi 115-20, Vietnam; 27 Department of Health Promotion, Faculty of Social and Behavioral Sciences, Hanoi University of Public Health, Hanoi 119-10, Vietnam

**Keywords:** hemodialysis, osteoporosis, fracture, health literacy, digital healthy diet literacy, comorbidities, biochemical parameters, detection, prevention, Vietnam

## Abstract

Osteoporosis is a common bone health disorder in hemodialysis patients that is linked with a higher morbidity and mortality rate. While previous studies have explored the associated factors of osteoporosis, there is a lack of studies investigating the impacts of health literacy (HL) and digital healthy diet literacy (DDL) on osteoporosis. Therefore, we aimed to investigate the associations of HL, DDL, and other factors with osteoporosis among hemodialysis patients. From July 2020 to March 2021, a cross-sectional study was conducted on 675 hemodialysis patients in eight hospitals in Vietnam. The data were collected by using the osteoporosis self-assessment tool for Asians (OSTA) and the 12-item short form of the health literacy questionnaire (HLS-SF12) on digital healthy diet literacy (DDL) and hemodialysis dietary knowledge (HDK). In addition, we also collected information about the socio-demographics, the clinical parameters, the biochemical parameters, and physical activity. Unadjusted and adjusted multinomial logistic regression models were utilized in order to investigate the associations. The proportion of patients at low, medium, and high levels of osteoporosis risk was 39.6%, 40.6%, and 19.8%, respectively. In the adjusted models, women had a higher likelihood of osteoporosis risk than men (odds ratio, OR, 3.46; 95% confidence interval, 95% CI, 1.86, 6.44; *p* < 0.001; and OR, 6.86; 95% CI, 2.96, 15.88; *p* < 0.001). The patients with rheumatoid arthritis (OR, 4.37; 95% CI, 1.67, 11.52; *p* = 0.003) and stomach ulcers (OR, 1.95; 95% CI, 1.01, 3.77; *p* = 0.048) were more likely to have a higher likelihood of osteoporosis risk than those without. The patients who had a higher waist circumference (WC), HL, and DDL were less likely to have a medium level of osteoporosis risk (OR, 0.95; 95% CI, 0.92, 0.98; *p* = 0.004; OR, 0.92; 95% CI, 0.88, 0.96; *p* < 0.001; OR, 0.96; 95% CI, 0.93, 0.99; *p* = 0.017, respectively) and a high level of osteoporosis risk (OR, 0.93; 95% CI, 0.89, 0.97; *p* = 0.001; OR, 0.89; 95% CI, 0.84, 0.94; *p* < 0.001; OR, 0.95; 95% CI, 0.91, 0.99; *p* = 0.008, respectively) compared with a low level of osteoporosis risk and to those with a lower WC, HL, and DDL. In addition, higher levels of hemoglobin (Hb) (OR, 0.79; 95% CI, 0.66, 0.95; *p* = 0.014), hematocrit (Hct) (OR, 0.95; 95% CI, 0.92, 0.99; *p* = 0.041), albumin (OR, 0.91; 95% CI, 0.83, 0.99; *p* = 0.030), and education (OR, 0.37; 95% CI, 0.16, 0.88; *p* = 0.025) were associated with a lower likelihood of a high level of osteoporosis risk. In conclusion, osteoporosis risk is highly prevalent in hemodialysis patients. Improved HL, DDL, education, WC, albumin, Hb, and Hct levels should be considered in preventing hemodialysis patients from developing osteoporosis.

## 1. Introduction

End-stage renal disease is a global health crisis [1,2]. The worldwide use of renal replacement therapies has increased remarkably from 2618 million people in 2010 to an estimated 5439 million people by 2030 [1]. Among the treatment modalities, hemodialysis is the most common in most countries, including Vietnam [2,3].

Osteoporosis is “a skeletal condition characterized by decreased density (mass/volume) of normally mineralized bone”, which leads to a high risk of fracture [4]. Osteoporosis is observed in a large number of hemodialysis patients, ranging from 23% to 41% [5,6,7]. The consequences of osteoporosis, such as the increased risk of fracture and the greater morbidity and mortality rate, have been documented in the literature [8,9]. Therefore, osteoporosis should be taken into account in the management of hemodialysis patients in order to reduce the burdens on the healthcare system and patients.

There are many factors that may contribute to an increased osteoporosis risk in chronic kidney patients, including abnormal biochemical markers (e.g., calcium, phosphorus, vitamin D, and parathyroid hormone), inflammatory cytokines status, sarcopenia, corticoid use, and lifestyle [10,11,12,13]. Factors that are associated with osteoporosis in pre-dialysis or hemodialysis patients have been found in previous studies, e.g., being female, being of an older age, and having a low body mass index [7,14,15]. Abdominal obesity was found to be a preventable factor for osteoporosis risk in hemodialysis patients [16].

Health literacy (HL) and digital healthy diet literacy (DDL) are defined as the individual’s capacity to find, understand, appraise, and apply the information that is related to health and digital-healthy-diet-related information in order to prevent disease, to improve healthy eating behavior, and to promote health outcomes [17,18]. The benefits of adequate health literacy in hemodialysis patients have been reported in prior studies, such as improved fluid management and psychological health [19,20]. However, there is limited information about the impacts of health literacy and digital healthy diet literacy on osteoporosis risk in hemodialysis patients. Meanwhile, insufficient health literacy has been associated with a lower adoption rate of osteoporosis-preventive behaviors [21], as well as a higher non-adherence to osteoporosis treatment [22].

Therefore, we conducted a multi-center study in order to explore the associated factors of osteoporosis in hemodialysis patients, where the roles of gender, comorbidities, biochemical parameters, HL, and DDL were emphasized. We hypothesized that the patients with higher HL and DDL, and greater levels in some biochemical components, had a lower likelihood of osteoporosis, whereas being female and living with comorbidities had an increased likelihood of osteoporosis.

## 2. Materials and Methods

### 2.1. Study Design and Sample

A cross-sectional study was conducted from July 2020 to March 2021. A total of 1048 eligible participants who were being treated at hemodialysis centers from eight hospitals in Vietnam were recruited, including Bai Chay Hospital (n = 81), Quang Ninh General Hospital (n = 103), Hai Phong International Hospital (n = 43), Hai Phong University of Medicine and Pharmacy Hospital (n = 82), Bach Mai Hospital (n = 251), Military Hospital 103 (n = 147), General Hospital of Agricultural(n = 171), and National Thai Nguyen Hospital (n = 170). A sample of 675 participants (who were aged 45 years or older, had received hemodialysis treatment for at least three months, and were able to communicate in Vietnamese) was analyzed.

### 2.2. Measurements

#### 2.2.1. The Risk of Osteoporosis

The osteoporosis self-assessment tool for Asians (OSTA) was used to evaluate the risk of osteoporosis. Koh et al. (2001) developed OSTA to screen the fracture risk among postmenopausal women in Asia. This self-assessment tool was not only applied to women, but also to men at risk of osteoporosis [23]. The OSTA score was calculated using Formula (1). Our present study used the dry weight of the participants to calculate the OSTA score.

OSTA score = [weight (kg) − age (year)] × 0.2(1)

The risk of osteoporosis was classified into three groups, in which a score > −1 presents a low risk, a score between −4 and −1 presents a medium risk, and a score < −4 presents a high risk [24].

The OSTA has been reported to be an acceptable tool in detecting the fracture risk and osteoporosis risk in hemodialysis patients [25]. It has also been tested in the Vietnamese population and has shown high sensitivity and specificity, at 74.6% and 81.4%, respectively [26].

#### 2.2.2. Socio-Demographics

The socio-demographic information was collected, including the age (year), gender (men vs. women), marital status (living with/without partner), educational status (illiterate or elementary, junior high school, and senior high school or above), occupation (working vs. not working), social status (low vs. medium or high), ability to pay for medication (very or fairly difficult vs. very or fairly easy), dry weight (kg), and hemodialysis vintage (years).

#### 2.2.3. Clinical Parameters

The clinical parameters were assessed, including comorbid conditions such as rheumatoid arthritis, stomach ulcers, and suspected COVID-19 symptoms (S-COVID19-S), waist circumference after dialysis (cm), and diuretic usage. The participants were screened and categorized as having S-COVID-19-S if they experienced any of the following symptoms: fever, cough, fatigue, dyspnea, myalgia, sputum production/expectoration, sore throat, running nose, confusion, headache, chest pain, rhinorrhea, diarrhea, and/or nausea/vomiting [27].

#### 2.2.4. Biochemical Parameters

The laboratory indicators were collected using chart review, including Hb (g/dL); mean corpuscular volume, MCV (fL); Hct (%); blood cells (white blood cells, 10^3^/μL; red blood cells, 10^6^/μL; platelet, 10^3^/μL); albumin (mg/dL); serum electrolytes (K^+^, mEq/L; Na^+^ and Cl^−^, mmol/L); phosphates (mg/dL); and parathyroid hormone (pg/mL).

#### 2.2.5. Physical Activities

The level of physical activity was measured using the international physical activity questionnaire, short version [28]. The tool was validated and used in Vietnam [20,29]. The patients rated their time spent on four daily activities, including sitting, walking, moderate, and vigorous physical activities throughout the last seven days. The overall physical activity score was calculated as per the instructions from previous studies [20,28].

#### 2.2.6. Health Literacy, Digital Healthy Diet Literacy, and Hemodialysis Diet Knowledge

The 12-item short form of the health literacy questionnaire (HLS-SF12) [30,31] and the digital healthy diet literacy (DDL-4) [18] were used to assess the HL and DDL of the participants. These scales have been validated and widely used in the Vietnamese population [18,30,32,33]. The participants chose one of four options to describe their difficulty for each question, in which, “1” was “very difficult” “2” was “fairly difficult”, “3” was “fairly easy”, and “4” was “very easy”. The HL and DDL indices were calculated using Formula (2). The higher index scores present the better HL or DDL levels [18,34].

Index = (Mean − 1) × (50/3)(2)

where *Index* is the specific index calculated, *Mean* is the mean of all participating items for each individual, 1 is the minimal possible value of the mean (leading to a minimum value of the index of 0), 3 is the range of the mean, and 50 is the chosen maximum value.

In addition, we used the hemodialysis dietary knowledge (HDK) scale to investigate the participants’ knowledge about the hemodialysis diet. This scale contains ten questions about protein, potassium, phosphorus, sodium, and water [35]. There are three possible options, including “correct,” “incorrect,” and “not sure”. The correct answer was categorized as “correct”, while incorrect or “not sure” answers were categorized as “incorrect”. The possible HDK score varies from 0 to 10, in which a higher score indicates better knowledge. The psychometric testing of HDK was conducted in a previous study [20].

### 2.3. Data Collection Procedure

The research assistants, including doctors, nurses, and medical students, collected the data after receiving a 4-h training session by two senior researchers at each hospital. Safety guidance for the prevention and control of COVID-19 was also provided during the training, including wearing masks, washing hands, and maintaining physical distance [36].

Participation in this study was completely voluntary. After receiving the consent forms, face-to-face interviews were conducted on patients at the hemodialysis departments. It took about 20–30 min to complete the questionnaire. Then, the research assistants collected information related to the clinical and biochemical parameters from medical records. Finally, all of the data were coded, cleaned, and analyzed confidentially by the researchers.

### 2.4. Data Analysis

Frequency and percentage were used for a categorical variable, while mean and standard deviation were used for a continuous variable using the chi-square test and one-way ANOVA appropriately. For the non-normal distributed variables, median and interquartile range were reported using the Kruskal–Wallis test. The unadjusted and adjusted models were performed by multinomial logistic regression to identify the associated factors of osteoporosis risks (using the low risk group as the reference). Only the factors with *p* < 0.20 in the unadjusted model were selected into the adjusted model [37]. In the adjusted model, each factor in each subgroup was adjusted by other factors in other subgroups. The correlations among the independent variables were checked prior to running the multinomial logistic regression to determine if these variables were highly correlated. The Spearman correlation coefficients of these variables that were less than 0.3 were accepted to be added to the adjusted model (Supplementary Table S1). All statistical analyses were performed using IBM SPSS Version 26.0 (IBM Corp., Armonk, NY, USA). Statistical significance was established at *p* < 0.05.

## 3. Results

### 3.1. Participants’ Socio-Demographics

The participants’ characteristics are described in Table 1. Of the 675 patients, 337 (50.1%) were male and 336 (49.9%) were female, with a mean age of 61.1 ± 9.6. Overall, the prevalence of osteoporosis risks at low, medium, and high levels were 39.6%, 40.6%, and 19.8%, respectively. The prevalence of low, medium, and high risks of osteoporosis was significantly different in some variables in the groups of socio-demographics (gender, education level, marital status, and medication payment ability); the clinical parameters (rheumatoid arthritis, waist circumference, and diuretic usage); the biochemical parameters (level of serum albumin); and the health literacy and digital health diet literacy (*p* < 0.05).

### 3.2. Associated Factors of Osteoporosis

Table 2 shows the results of the unadjusted and the adjusted multinomial logistic regression models. In the adjusted model, a medium osteoporosis risk (OR, 3.46; 95% CI, 1.86, 6.44; *p* < 0.001) and a high osteoporosis risk (OR, 6.86; 95% CI, 2.96, 15.88; *p* < 0.001) were more likely in the female participants. The participants who were at a high risk of osteoporosis were more likely to have rheumatoid arthritis (OR, 4.37; 95% CI, 1.67, 11.52; *p* = 0.003), while those at a medium risk of osteoporosis were more likely to have stomach ulcers (OR, 1.95; 95% CI, 1.01, 3.77; *p* = 0.048). The odds of a medium osteoporosis risk decreased by 5%, 8%, and 4% for each increment by one unit in waist circumference (OR, 0.95; 95% CI, 0.92, 0.98; *p* = 0.004), HL (OR, 0.92; 95% CI, 0.88, 0.96; *p* < 0.001), and DDL (OR, 0.96; 95% CI, 0.93, 0.99; *p* = 0.017), respectively. The odds of a high osteoporosis risk declined by 7%, 21%, 5%, 9%, 11%, and 5% for each increment by one unit in waist circumference (OR, 0.93; 95% CI, 0.89, 0.97; *p* = 0.001), Hb level (OR, 0.79; 95% CI, 0.66, 0.95; *p* = 0.014), Hct (OR, 0.95; 95% CI, 0.92, 0.99; *p* = 0.041), albumin level (OR, 0.91; 95% CI, 0.83, 0.99; *p* = 0.030), HL (OR, 0.89; 95% CI, 0.84, 0.94; *p* < 0.001), and DDL (OR, 0.95; 95% CI, 0.91, 0.99; *p* = 0.008), respectively. The patients with a junior high school level of education had a lower likelihood of having osteoporosis risk compared to those who were illiterate or at the elementary level of education (OR, 0.37; 95% CI, 0.16, 0.88; *p* = 0.025).

## 4. Discussion

Using a self-assessment tool, more than 60% of our hemodialysis patients presented a medium and high level of osteoporosis risk. The high prevalence of osteoporosis in hemodialysis patients has been reported in previous studies that used different diagnostic methods, such as an ultrasound bone densitometry [38], a bone density scan [5,6], and a bone histomorphometry [39]. The OSTA has been suggested for early osteoporosis detection in the healthy population [26] or in individuals with type 2 diabetes mellitus [40]. It is also capable of distinguishing the fracture status in hemodialysis patients [25]. This screening tool may help physicians to prioritize the high-risk patients for further diagnosis and for specific strategies to manage those patients.

In this study, we found that higher HL, DDL, and waist circumference, and higher levels of Hb, Hct, albumin, and education, protected our participants from osteoporosis risk, while being female and having comorbid diseases were associated with a higher likelihood of having bone health disorder.

The most important finding in this study is that higher HL and DDL were associated with a lower likelihood of osteoporosis risk in patients who were undergoing hemodialysis. Similarly, our patients with a higher education level had lower odds of osteoporosis, which is in line with another study’s findings [41]. In fact, the promotion of healthy habits, a balanced nutrient intake, and regular exercise is highly recommended in order to reduce the risk of osteoporosis [42,43]. People with higher HL and DDL scores tend to have better eating behaviors [18,44]. However, the level of HL among hemodialysis patient was insufficient [45]. Nowadays, numerous resources, such as the Internet, media, books, etc., can provide hemodialysis patients with information about healthy eating; however, not all of these methods are qualified. Thus, particular attention should be given to such interventions in order to improve patients’ health and healthy diet literacy. For example, these patients should be educated and suggested formal websites in order to help them with adequate literacy about the hemodialysis diet.

Another important finding in the present study is that the waist circumference protected our participants from the risk of osteoporosis. This finding is congruent with a previous study that supported a lower risk of osteoporosis in hemodialysis patients with abdominal obesity [16]. A higher waist circumference reflects abdominal obesity, which may increase bone density by stimulating bone growth and improving the bones’ weight tolerance [46]. However, other studies have reported that people with a higher waist circumference/abdominal obesity have a higher risk of osteoporosis/fractures or a worse bone mineral density [47,48,49]. The inconsistency between these results may be due to the difference between the studies in the measuring methods, the fracture sites, the covariates, and the waist circumference classification. Thus, further studies assessing the relationship between waist circumference and osteoporosis risk among hemodialysis patients are needed.

Remarkably, in this study, higher serum albumin was significantly associated with lower odds of osteoporosis risk. In line with our findings, another study on rheumatoid arthritis patients during the postmenopausal stage revealed that the serum albumin concentrations were lower in the osteoporosis group than in the non-osteoporosis group [50]. Likewise, hypoalbuminemia was associated with a higher risk of osteoporosis and future fractures [51,52]. A possible explanation for this association is that low levels of serum albumin may directly activate osteoclasts and inhibit osteogenesis through its link with the nuclear factor-κB [53]. Moreover, our study found that higher Hb and Hct levels were protective factors against osteoporosis. This concurs with other studies [54,55,56]. In fact, lower levels of Hb and Hct reflect an anemia status, which increases osteoclast activity [55]. Hence, in clinical practice, health care providers should be aware of the correction between albumin, Hb, and Hct levels in order to protect against osteoporosis in hemodialysis patients.

Inversely, being female was associated with an increased level of osteoporosis risk in our participants. It has been indicated consistently in the literature that women have a higher risk of osteoporosis than men [7,57]. Bone loss may be due to a deficiency in estrogen, which is highly prevalent in menopausal women [58]. However, other studies have found that decreased estrogen levels play a major role in osteoporosis development for both sexes [59,60]. Therefore, although female patients receive more attention in early osteoporosis detection, osteoporosis in men should also be taken into account.

Our findings have also indicated that hemodialysis patients who have comorbid diseases tend to have a higher level of osteoporosis risk. Patients who are diagnosed with rheumatoid arthritis have a higher risk of osteoporosis, which is possibly due to their inflammation status, cytokine release, and autoantibody production [61]. Meanwhile, persistent inflammation in chronic kidney disease is obviously present [62]. Notably, having stomach ulcers was another risk factor of osteoporosis in our participants. This finding is consistent with the findings from previous studies [63,64]. A possible explanation for this association is that patients with peptic ulcers are usually treated with omeprazole, which has a negative association with the risk of vertebral fractures [65]. Therefore, early diagnosis and the establishment and implementation of appropriate alternative treatments for hemodialysis patients with other diseases could reduce osteoporosis risk.

This study has some limitations. Firstly, the causality of the protective factors on osteoporosis was not able to be confirmed in a cross-sectional study design. Further experimental studies are suggested in order to examine the effects of the biochemical components and health and diet literacy on osteoporosis in hemodialysis patients. Secondly, the biochemical parameters (e.g., vitamin D, calcium, and liver enzymes) that may contribute to osteoporosis have not been investigated in this study due to insufficient data. Thirdly, the OSTA score was intended to reflect the osteoporosis risk or the fracture risk instead of confirmed osteoporosis status. Therefore, the results should be interpreted with caution. The use of a combination of osteoporosis diagnosis methods (e.g., using bone biopsy or biochemical parameters) is suggested in future research and clinical practice. Despite the limitations, OSTA may be an acceptable solution in detecting the osteoporosis risk in some contexts, particularly those regarding issues such as the trained technicians required, high cost, and instrument availability.

## 5. Conclusions

In this study, we have found that female patients and those with comorbid conditions were more likely to have osteoporosis/fracture risk. Meanwhile, patients with higher health and diet literacy, education, waist circumference, and greater levels of some biochemical components (albumin, Hb, and Hct) had a lower likelihood of osteoporosis risk. The results suggest that these protective factors should be a consideration in future interventions in order to prevent osteoporosis/fracture risk in hemodialysis patients.

## Figures and Tables

**Table 1:** Patients’ characteristics and OSTA (n = 675).

Variables	Total n (%)	OSTA			*p*
		Low (n;% = 267; 39.6)	Medium (n;% = 274; 40.6)	High (n;% = 134; 19.8)	
**Socio-demographics**					
Gender					<0.001 ^a^
Male	337 (50.1)	176 (65.9)	119 (43.6)	42 (31.6)	
Female	336 (49.9)	91 (34.1)	154 (56.4)	91 (68.4)	
Education					0.001 ^a^
Illiterate or elementary	316 (50.5)	101 (41.4)	142 (55.7)	73 (57.5)	
Junior high school	182 (29.1)	91 (31.3)	67 (26.3)	24 (18.9)	
Senior high school or above	128(20.4)	52 (21.3)	46 (18.0)	30 (23.6)	
Working status					0.660 ^a^
Not working	249 (36.9)	93 (34.8)	104 (38.0)	52.0 (38.8)	
Working	426 (63.1)	174 (65.2)	170 (62.0)	82 (61.2)	
Marital status					0.009 ^a^
Without partner	52 (7.8)	13 (4.9)	21 (7.8)	18 (13.6)	
With partner	616 (92.2)	253 (95.1)	249 (92.2)	114 (86.4)	
Social status					0.318 ^a^
Low	198 (29.3)	70 (26.2)	88 (32.1)	40 (29.9)	
Middle and high	477 (70.7)	197 (73.8)	186 (67.9)	94 (70.1)	
Medication payment ability					0.035 ^a^
Very or fairly difficult	521 (782)	219 (82.3)	209 (77.7)	93 (71.0)	
Very or fairly easy	145 (21.8)	47 (17.7)	60 (22.3)	38 (29.0)	
Hemodialysis vintage, years (Median, IQR)	4.4 (2.2, 7.9)	4.4 (2.4, 7.9)	4.4 (2.4, 8.2)	4.2 (2.0, 7.9)	0.966 ^b^
**Clinical Parameters**					
S-COVID-19-S					0.057 ^a^
Without S-COVID-19-S	204 (30.2)	94 (35.2)	77 (28.1)	33 (24.6)	
With S-COVID-19-S	471 (69.8)	173 (64.8)	197 (71.9)	101 (74.4)	
Rheumatoid arthritis					<0.001 ^a^
No	606 (89.8)	251 (94.0)	248 (90.5)	107 (79.9)	
Yes	69 (10.2)	16 (6.0)	26 (9.5)	27 (20.1)	
Stomach ulcers					0.068 ^a^
No	538 (79.7)	224 (83.9)	208 (75.9)	106 (79.1)	
Yes	137 (20.3)	43 (16.1)	66 (24.1)	28 (20.9)	
Diuretic usage					0.002 ^a^
No	351 (73.1)	156 (81.3)	135 (69.9)	60 (63.2)	
Yes	129 (26.9)	36 (18.8)	58 (30.1)	35 (36.8)	
HBsAg					0.853 ^a^
Negative	425 (91.6)	164 (91.6)	170 (90.9)	91 (92.9)	
Positive	39 (8.4)	15 (8.4)	17 (9.1)	7 (7.1)	
HCV					0.678 ^a^
Negative	330 (71.1)	127 (70.9)	130 (69.5)	73 (74.5)	
Positive	134 (28.9)	52 (29.1)	57 (30.5)	25 (25.5)	
WC, cm (Mean ± SD)	76.9 ± 9.9	78.9 ± 9.7	75.7 ± 9.8	75.5 ±10.4	0.001 ^c^
**Biochemical parameters**					
Hb, g/dL (Median, IQR)	9.7 (8.4, 11.1)	9.8 (8.6, 11.1)	9.8 (8.3, 11.3)	9.4 (7.9, 10.7)	0.064 ^b^
WBC, 10^3^/μL (Median, IQR)	5.9 (4.5, 7.3)	5.8 (4.5, 7.0)	6.0 (4.5, 7.5)	6.0 (4.6, 7.4)	0.606 ^b^
RBC, 10^6^/μL (Median, IQR)	3.3 (2.7, 3.7)	3.3 (2.8, 3.8)	3.3 (2.7, 3.8)	3.1 (2.7, 3.7)	0.593 ^b^
MCV, fL (Median, IQR)	90.0 (83.0, 94.6)	89.7 (83.4, 94.2)	89.9 (80.7, 94.1)	91.6 (85.3, 95.9)	0.099 ^b^
Hct, % (Mean ± SD)	30.0 ± 5.8	30.1 ± 5.3	30.5 ± 6.3	28.9 ± 5.7	0.095 ^c^
PLT, 10^3^/μL (Median, IQR)	205.0 (163.0, 245.0)	197.0 (163.0, 236.0)	206.0 (168.0, 252.0)	208.0 (157.0, 251.0)	0.200 ^b^
PO_4_, mg/dL (Median, IQR)	1.8 (1.4, 2.6)	1.8 (1.4, 2.4)	1.7 (1.4, 2.8)	2.0 (1.3, 3.4)	0.800^b^
PTH, pg/mL (Median, IQR)	23.9 (16.4, 45.8)	23.4 (16.5, 43.5)	25.2 (14.1, 46.5)	25.2 (19.1, 51.0)	0.568 ^b^
K^+^, mEq/L (Mean ± SD)	4.2 ± 0.9	4.2 ± 0.9	4.3 ± 0.9	4.2 ± 0.8	0.817 ^c^
Na^+^, mmol/L (Mean ± SD)	135.9 ± 4.7	136.3 ± 3.1	135.7 ± 5.3	135.9 ± 5.9	0.518 ^c^
Cl^−^, mmol/L (Mean ± SD)	97.8 ± 5.1	97.3 ± 4.4	97.9 ± 5.9	98.5 ± 4.7	0.193 ^c^
Albumin, mg/dL (Mean ± SD)	38.7 ± 5.6	39.3 ± 6.3	38.7 ± 4.9	37.3 ± 5.6	0.047 ^c^
**Physical activity**, MET-min/wk					0.895 ^a^
Tertile 1 (MET ≤ 180)	168 (33.5)	67 (34.5)	68 (33.0)	33 (32.4)	
Tertile 2 (180 < MET ≤ 723)	164 (32.7)	59 (30.4)	72 (35.0)	33 (32.4)	
Tertile 3 (MET > 723)	170 (33.9)	68 (35.1)	66 (32.0)	36 (35.3)	
**Health/diet literacy and diet knowledge**					
HL index (Mean ± SD)	25.2 ± 9.2	25.6 ± 8.6	22.9 ± 9.3	22.2 ± 9.3	<0.001 ^c^
DDL index (Mean ± SD)	24.1 ± 11.4	25.1 ± 11.1	21.2 ± 11.3	20.6 ± 1.7	<0.001 ^c^
HDK (Mean ± SD)	5.4 ± 2.5	5.2 ± 2.5	5.3 ± 2.4	5.3 ± 2.6	0.788 ^c^

Abbreviation: SD, standard deviation; IQR, interquartile range; S-COVID-19-S, suspected COVID-19 symptoms; HBsAg, hepatitis B surface antigen; HCV, hepatitis C virus; WC, waist circumference; Hb, hemoglobin; WBC, white blood cell; RBC, red blood cell; MCV, mean corpuscular volume; Hct, hematocrit; PLT, platelet count; PO4, phosphates; PTH, parathyroid hormone; K^+^, kali; Na^+^, natri; Cl^−^, clo; MET-min/wk, metabolic equivalent task scored in minutes per week; HL, health literacy; DDL, digital health diet literacy; HDK, hemodialysis dietary knowledge; OSTA, osteoporosis self-assessment tool for Asians. ^a^ Result of the chi-square test; ^b^ Result of Kruskal–Wallis test; ^c^ Result of one-way ANOVA.

**Table 2:** Associated factors of osteoporosis via unadjusted and adjusted multinominal logistic regression analysis.

Associated Factors	Medium Risk of Osteoporosis *				High Risk of Osteoporosis *			
	Unadjusted Model		Adjusted Model		Unadjusted Model		Adjusted Model	
	OR (95% CI)	*p*	OR (95% CI)	*p*	OR (95% CI)	*p*	OR (95% CI)	*p*
**Socio-demographics**								
Gender			1.00					
Male	1.00				1.00		1.00	
Female	2.50 (1.77, 3.55)	<0.001	3.46 (1.86, 6.44)	<0.001 ^a^	4.19 (2.69, 6.54)	<0.001	6.86 (2.96, 15.88)	<0.001 ^a^
Education								
Illiterate or elementary	1.00		1.00		1.00		1.00	
Junior high school	0.52 (0.35, 0.79)	0.002	0.63 (0.33, 1.20)	0.158 ^a^	0.37 (0.21, 0.63)	<0.001	0.37 (0.16, 0.88)	0.025 ^a^
Senior high school or above	0.63 (0.39, 1.01)	0.054	1.02 (0.41, 2.53)	0.963 ^a^	0.80 (0.47, 1.37)	0.414	0.82 (0.27, 2.53)	0.731 ^a^
Working status								
Not working	1.00				1.00			
Working	0.87 (0.62, 1.24)	0.450			0.84 (0.55, 1.29)	0.435		
Marital status								
Without partner	1.00				1.00		1.00	
With partner	0.61 (0.30, 1.24)	0.173	0.82 (0.09, 6.97)	0.817 ^a^	0.33 (0.15, 0.69)	0.003	0.60 (0.06, 6.46)	0.674 ^a^
Social status								
Low	1.00		1.00		1.00		1.00	
Middle and high	0.75 (0.52, 0.09)	0.132	1.15 (0.60, 2.23)	0.673 ^a^	0.84 (0.53, 1.32)	0.442	1.07 (0.46, 2.50)	0.874 ^a^
Medication payment ability								
Very or fairly difficult	1.00		1.00		1.00		1.00	
Very or fairly easy	1.34 (0.87, 2.05)	0.181	1.63 (0.69, 3.84)	0.265 ^a^	1.90 (1.16, 3.11)	0.010	0.98 (0.32, 2.97)	0.968 ^a^
Hemodialysis vintage, years	1.01 (0.97, 1.05)	0.620			0.99 (0.94, 1.04)	0.609		
**Clinical parameters**								
S-COVID-19-S								
Without S-COVID-19-S	1.00		1.00		1.00		1.00	
With S-COVID-19-S	1.39 (0.97, 2.00)	0.076	1.18 (0.62, 2.24)	0.620 ^b^	1.66 (1.04, 2.65)	0.033	2.37 (0.93, 6.07)	0.071 ^b^
Rheumatoid arthritis								
No	1.00		1.00		1.00		1.00	
Yes	1.65 (0.86, 3.14)	0.132	0.92 (0.37, 2.33)	0.865 ^b^	3.96 (2.05, 7.65)	<0.001	4.37 (1.67, 11.52)	0.003 ^b^
Stomach ulcers								
No	1.00		1.00		1.00		1.00	
Yes	1.65 (1.08, 2.54)	0.021	1.95 (1.01, 3.77)	0.048 ^b^	1.38 (0.81, 2.34)	0.237	1.53 (0.66, 3.54)	0.322 ^b^
Diuretic usage								
No	1.00		1.00		1.00		1.00	
Yes	1.86 (1.16, 2.99)	0.010	1.22 (0.63, 2.34)	0.562 ^b^	2.53 (1.46, 4.39)	0.001	2.19 (0.99, 4.86)	0.053 ^b^
HBsAg								
Negative	1.00				1.00			
Positive	1.09 (0.53, 2.26)	0.810			0.84 (0.33, 2.14)	0.716		
HCV								
Negative	1.00				1.00			
Positive	1.07 (0.68, 1.68)	0.765			0.84 (0.48, 1.46)	0.530		
WC	0.97 (0.95, 0.99)	0.001	0.95 (0.92, 0.98)	0.004 ^b^	0.97 (0.94, 0.99)	0.008	0.93 (0.89, 0.97)	0.001 ^b^
**Biochemical parameters**								
Hb	0.99 (0.87, 1.09)	0.779	0.98 (0.86, 1.13)	0.802 ^c^	0.85 (0.75, 0.97)	0.017	0.79 (0.66, 0.95)	0.014 ^c^
WBC	1.05 (0.97, 1.14)	0.248			1.06 (0.96, 1.18)	0.266		
RBC	1.07 (0.85, 1.35)	0.569			0.89 (0.66, 1,21)	0.465		
MCV	0.99 (0.98, 1.01)	0.814	0.99 (0.97, 1.01)	0.411 ^c^	1.02 (0.99, 1.04)	0.124	1.01 (0.98, 1.04)	0.549 ^c^
Hct	1.01 (0.98, 1.05)	0.592	1.01 (0.97, 1.06)	0.679 ^c^	0.96 (0.92, 1.01)	0.089	0.95 (0.92, 0.99)	0.041 ^c^
PLT	1.01 (0.99, 1.01)	0.221			1.01 (0.99, 1.01)	0.441		
PO_4_	1.08 (0.89, 1.32)	0.435			1.06 (0.84, 1.35)	0.610		
PTH	1.01 (0.98, 1.02)	0.876			1.01 (0.99, 1.03)	0.311		
K^+^	1.09 (0.84, 1.40)	0.524			1.05 (0.77, 1.43)	0.768		
Na^+^	0.97 (0.92, 1.02)	0.257			0.98 (0.92, 1.04)	0.506		
Cl^−^	1.03 (0.98, 1.08)	0.200	1.02 (0.96, 1.07)	0.538 ^c^	1.05 (0.99, 1.11)	0.083	1.02 (0.96, 1.10)	0.503 ^c^
Albumin	0.98 (0.96, 1.02)	0.395	0.98 (0.91, 1.05)	0.286 ^c^	0.93 (0.88, 0.98)	0.012	0.91 (0.83, 0.99)	0.030 ^c^
**Physical activity**								
Tertile 1 (MET ≤ 180)	1.00				1.00			
Tertile 2 (180 < MET ≤ 723)	1.20 (0.74, 1.95)	0.454			1.14 (0.63, 2.06)	0.676		
Tertile 3 (MET > 723)	0.96 (0.59, 1.54)	0.855			1.08 (0.60, 1.92)	0.807		
**Health/diet literacy and diet knowledge**								
HL index	0.97 (0.95, 0.99)	0.001	0.92 (0.88, 0.96)	<0.001 ^d^	0.96 (0.94, 0.98)	0.001	0.89 (0.84, 0.94)	<0.001 ^c^
DDL index	0.97 (0.95, 0.98)	<0.001	0.96 (0.93, 0.99)	0.017 ^d^	0.97 (0.95, 0.98)	<0.001	0.95 (0.91, 0.99)	0.008 ^c^
HDK	1.02 (0.95, 1.09)	0.668			1.03 (0.95, 1.12)	0.502		

Abbreviation: OR, odds ratio; CI, confidence interval; SD, standard deviation; S-COVID-19-S, suspected COVID-19 symptoms; HBsAg, hepatitis B surface antigen; HCV, hepatitis C virus; WC, waist circumference; Hb, hemoglobin; WBC, white blood cell; RBC, red blood cell; MCV, mean corpuscular volume; Hct, hematocrit; PLT, platelet count; PO_4_, phosphates; PTH, parathyroid hormone; K^+^, kali; Na^+^, natri; Cl^−^, clo; HL, health literacy; DDL, digital health diet literacy; HDK, hemodialysis dietary knowledge; OSTA, osteoporosis self-assessment tool for Asians. * Low risk of osteoporosis is the reference group. ^a^ the adjusted multinominal logistic regression, which adjusted for variables (*p* < 0.2) in the groups of clinical parameters, biochemical parameters, health/diet literacy and diet knowledge; ^b^ the adjusted multinominal logistic regression, which adjusted for variables (*p* < 0.2) in the groups of socio-demographics, biochemical parameters, health/diet literacy and diet knowledge; ^c^ the adjusted multinominal logistic regression, which adjusted for variables (*p* < 0.2) in the groups of socio-demographics, clinical parameters, health/diet literacy and diet knowledge; ^d^ the adjusted multinominal logistic regression, which adjusted for variables (*p* < 0.2) in the groups of socio-demographics, clinical parameters, and biochemical parameters.

## Data Availability

Data will be available upon reasonable request to the corresponding author.

## Supplementary Materials

The following supporting information can be downloaded at: https://www.mdpi.com/article/10.3390/nu14235122/s1, Table S1: The correlations between independent variables.
