# Supplementary material for: Osteoporosis Risk in Hemodialysis Patients: The Roles of Gender, Comorbidities, Biochemical Parameters, Health and Diet Literacy
Source: Nutrients. 2022 Dec 2;14(23):5122. doi: 10.3390/nu14235122 (PMC9741163; doi:10.3390/nu14235122)
Supplement: Supplementary file 1 [file nutrients-14-05122-s001.zip › nutrients-2041271-supplementary.pdf]

Table S1: The correlations between independent variables

	Gender	Education level	Marital status	Social status	Payment ability	S-COVID19-S	Rheumatoid arthritis	Stomach ulcers	Diuretic use	WC	Hb	MCV	Hct	Cl	Albumin	HL	DDL
Gender	1.000																
Education level	-.103**	1.000															
Marital status	-.146**	.107**	1.000														
Social status	-.053	.302**	.120**	1.000													
Payment ability	-.024	.155**	.031	.246**	1.000												
S-COVID19-S	.138**	.057	.029	-.091*	-.180**	1.000											
Rheumatoid arthritis	.074	.056	-.030	-.105**	-.012	.158**	1.000										
Stomach ulcers	-.003	-.030	-.007	-.071	-.042	.172**	.207**	1.000									
Diuretic use	.007	-.146**	.063	.004	.101*	.111*	.172**	.001	1.000								
WC	-.101*	.077	.052	-.001	.016	-.053	.163**	.076	.118*	1.000							
Hb	.064	.045	-.003	-.089	-.120**	.019	.081	.063	-.018	.110*	1.000						
MCV	.050	-.031	-.023	.058	.057	-.162**	.021	.114**	.045	.370**	.098*	1.000					
Hct	.095*	.057	.009	-.096*	-.102*	.040	.105*	.061	-.019	.076	.944**	.001	1.000				
Cl	-.010	.042	-.097	-.162**	.090	.072	.156**	-.002	.064	-.113*	-.180**	-.187**	-.092	1.000			
Albumin	.093	-.130*	.032	.070	-.024	.003	-.082	-.067	.029	-.135*	.186**	-.056	.181**	-.287**	1.000		
HL	-.024	.151**	.038	.131**	.131**	.030	-.129**	-.094*	-.056	-.138**	-.040	-.069	-.028	.042	-.051	1.000	
DDL	-.075	.213**	.040	.041	.069	.044	-.011	-.025	-.061	.001	.013	.051	.008	-.015	-.036	.740**	1.000

Abbreviation: S-COVID-19-S, suspected COVID-19 symptoms; WC, waist circumference; Hb, hemoglobin; MCV, mean corpuscular volume; Hct, hematocrit; Cl-, clo; HL, health literacy; DDL, digital health diet literacy
